# The Impact of Programmed Death-Ligand 1 Expression on the Prognosis of Early Stage Resected Non-Small Cell Lung Cancer: A Meta-Analysis of Literatures

**DOI:** 10.3389/fonc.2021.567978

**Published:** 2021-02-23

**Authors:** Tao Shi, Shuai Zhu, Hengjuan Guo, Xiongfei Li, Shikang Zhao, Yanye Wang, Xi Lei, Dingzhi Huang, Ling Peng, Ziming Li, Song Xu

**Affiliations:** ^1^ Precision Medicine Center, Tianjin Medical University General Hospital, Tianjin, China; ^2^ Department of Lung Cancer Surgery, Tianjin Key Laboratory of Lung Cancer Metastasis and Tumor Microenvironment, Lung Cancer Institute, Tianjin Medical University General Hospital, Tianjin, China; ^3^ Department of Respiratory and Critical Care, Tianjin Medical University General Hospital, Tianjin, China; ^4^ Department of Thoracic Oncology, Tianjin Medical University Cancer Institute and Hospital, Tianjin, China; ^5^ Department of Radiotherapy, The First Affiliated Hospital, School of Medicine, Zhejiang University, Hangzhou, China; ^6^ Shanghai Lung Cancer Center, Shanghai Chest Hospital, Shanghai Jiao Tong University, Shanghai, China

**Keywords:** PD-L1, NSCLC, meta-analysis, prognosis, resection

## Abstract

**Introduction:**

Previous studies have demonstrated that programmed cell death-ligand 1 (PD-L1) serves as biomarker for poor prognosis and survival in advanced-stage non-small cell lung cancer (NSCLC) patients. However, the merit of PD-L1 expression to predict the prognosis of early stage NSCLC patients who underwent complete resection remains controversial. In the present study, we performed a meta-analysis to investigate the relationship between PD-L1 expression and prognosis in patients with early stage resected NSCLC.

**Methods:**

Electronic databases, including PubMed, EMBASE, and the Cochrane Library, were searched until July 23 2020 for studies evaluating the expression of PD-L1 and the prognosis of resected NSCLCs. Hazard ratios (HRs) with 95% confidence intervals (CIs) of overall survival (OS) and disease-free survival (DFS) were pooled and analyzed. Heterogeneity and publication bias analyses were also assessed.

**Results:**

A total of 15 studies involving 3,790 patients were considered in the present meta-analysis. The pooled HR indicated that PD-L1 expression related to a much shorter DFS (HR = 1.56, 95% CI: 1.18–2.05, p < 0.01), as well a significantly worse OS (HR = 1.68, 95% CI: 1.29–2.18, p < 0.01). Furthermore, our analysis indicated that PD-L1 expression was significantly associated with gender (male *vs.* female: OR = 1.27, 95% CI:1.01–1.59, p = 0.038), histology (ADC *vs*. SCC: OR = 0.54, 95% CI:0.38–0.77, p = 0.001), TNM stage (I *vs*. II–III: OR = 0.45, 95% CI:0.34–0.60, p = 0.000), smoking status (Yes *vs* No: OR = 1.43, 95% CI:1.14–1.80, p = 0.002) and lymph node metastasis (N+ *vs* N−: OR = 1.97, 95% CI:1.26–3.08, p = 0.003).

**Conclusions:**

The results of this meta-analysis suggest that PD-L1 expression predicts an unfavorable prognosis in early stage resected NSCLCs. The role of personalized anti-PD-L1/PD-1 immunotherapy in the adjuvant settings of resected NSCLC warrants further investigation.

## Introduction

Lung cancer is the most commonly diagnosed cancer and a leading cause of cancer-related deaths (1). Surgery is the standard treatment for early stage non-small cell lung cancer (NSCLC); however, postoperative prognosis remains unsatisfactory, with a 5-year survival rate ranging between 71 and 83% (2).Therefore, it is essential to identify new biomarkers for efficient clinical decision making and improve patient outcomes. Currently, blockade of the programmed cell death 1 (PD-1)/PD-1 ligand 1 (PD-L1) signaling pathway is one of the most promising immunotherapeutic strategies in boosting the immune system in the fight against cancer (3, 4). Programmed death 1 (PD-1), an important immune checkpoint molecule, is an immune-inhibitory receptor expressed on the surface of activated T cells in response to persistent inflammatory stimuli (5, 6). PD-L1 expressed on the tumor cells binds to the PD-1 receptors on activated T cells, resulting in the inhibition of the cytotoxic T cells. Blockade of the PD-1/PD-L1 pathway with monoclonal antibodies is a promising therapeutic strategy, with prominent clinical benefits of this checkpoint-blockade observed in recent clinical trials (7, 8).

Previously, a study has demonstrated that PD-L1 is a marker of poor prognosis and survival in advanced-stage NSCLC patients (9). A meta-analysis, performed on six studies with 1,157 patients, demonstrated that NSCLC patients with positive PD-L1 expression exhibited a much poorer OS (10). Another meta-analysis by Li et al., which included the largest number of patients (fifty studies with 11,383 patients), also indicated that PD-L1 IHC expression was related to poor overall survival (11). However, the searching deadline for Li’s study was January 2018, and more recent studies focusing on PD-L1 and prognosis in NSCLC were missing. More importantly, all of previous studies, including Li’s study, were performed on the NSCLC patients of both early and advanced stages, and they only took OS into account. Therefore, the impact of PD-L1 expression in the prognosis of patients with early stage NSCLCs following complete resection remains controversial. In the present study, we performed a meta-analysis of all available evidence not only to analyze OS but also assess the correlation between PD-L1 expression and DFS in early stage surgically resected NSCLC patients, which is more accurate and valuable to reflect the influence of PD-L1 expression on the survival of NSCLC.

## Methods

This study was reported on the basis of the Preferred Reporting Items for Systematic Reviews and Meta-Analyses (PRISMA) statement guidelines (**Supplementary Data 1**).

Relevant studies were retrieved by searching PubMed, Embase, and the Central Registry of Controlled Trials of the Cochrane Library, using the following terms: “Carcinoma, Non-Small Cell Lung” AND “PD-L1 protein, human” AND “Prognosis”. (**Supplementary Data 2**) The final search period was July 23 2020. Two authors (SX and SZ) performed the search independently. We restricted our research to studies published in English.

Eligible studies were in agreement with the following criteria: (1) the histology type of cancer was NSCLC; (2) valid TNM stage and cancer differentiation, as well as sufficient survival data, such as hazard ratio (HR) with 95% confidence intervals (CI), OS, and DFS were available (3) were published in English; (4) evaluated the association between PD-L1 expression and prognosis or pathological features; (5) involved early stage resectable NSCLC patients; (6) had similar research experimental design and methods; (7) PD-L1 expression was divided into high (positive) and low (negative) categories; and (8) relevant information could be extracted from the full-text study. Exclusion criteria included: (1) duplicate reports, ongoing studies, letters, conference papers, and reviews; (2) studies regarding lung cancer cell lines, animal models, and other types of cancer; (3) studies with insufficient survival data for which HR and CI could not be determined; (4) papers not published in English; (5) methods and experimental design differed from those of the selected studies.

The Newcastle–Ottawa quality assessment scale (NOS) and National Institute for Clinical Excellence (NICE) quality assessment scale was performed to assess methodological quality and risk of bias for cohort studies and case series studies, respectively. The primary outcomes of our study were disease-free survival (DFS) and overall survival (OS). The characteristic details of the publications, including the first author’s name, publication year, tumor type, PD-L1 level, stage, the evaluation method of the PD-L1expression, were extracted by two independent investigators. Any disagreement was discussed between investigators to reach a consensus. Multivariate analysis results were extracted as some included studies performed univariate analysis. We used the data directly from the included studies, providing precise HR (95% CI). In the case of studies only providing Kaplan–Meier survival curves, the Engauge Digitizer version 2.11 software was used to extract relevant numerical values from survival curves and calculate the HR (95% CI) (12, 13).

The STATA 15.0 (Stata Corporation, College Station, USA) software and R package 4.0.2 were used for data synthesis and analysis.

The random-effects model was employed in case of potential heterogeneity and to avoid underestimation of standard errors of pooled estimates in this meta-analysis. HRs for DFS and OS with 95% CIs according to the expression status of PD-L1 were pooled. The pooled odds ratios (ORs) were used to investigate the correlation between PD-L1 expression and clinicopathological features.

The heterogeneity test was performed using the Cochrane’s Q test (Chi-squared test; Chi^2^) and I^2^ metric (14). A chi-squared P value <0.1 or an I^2^ statistic >50% was defined as statistically significant heterogeneity (15). Moreover, the potential publication bias was assessed through Begg’s funnel plots (16). P <0.05 was considered as statistically significant based on the two-sided test. Subgroup analysis was conducted according to gender, histology, TNM stage, smoking status, and lymph node metastasis. An HR >1 reflected longer OS or DFS for PD-L1 negative patients.

Sensitivity analyses (17) were conducted to investigate the influence of a specific study on the pooled risk estimate by removing one study in each turn (**Figures 2D**, **3D**, **Supplementary Data 3**).

## Results

**Figure 1:**
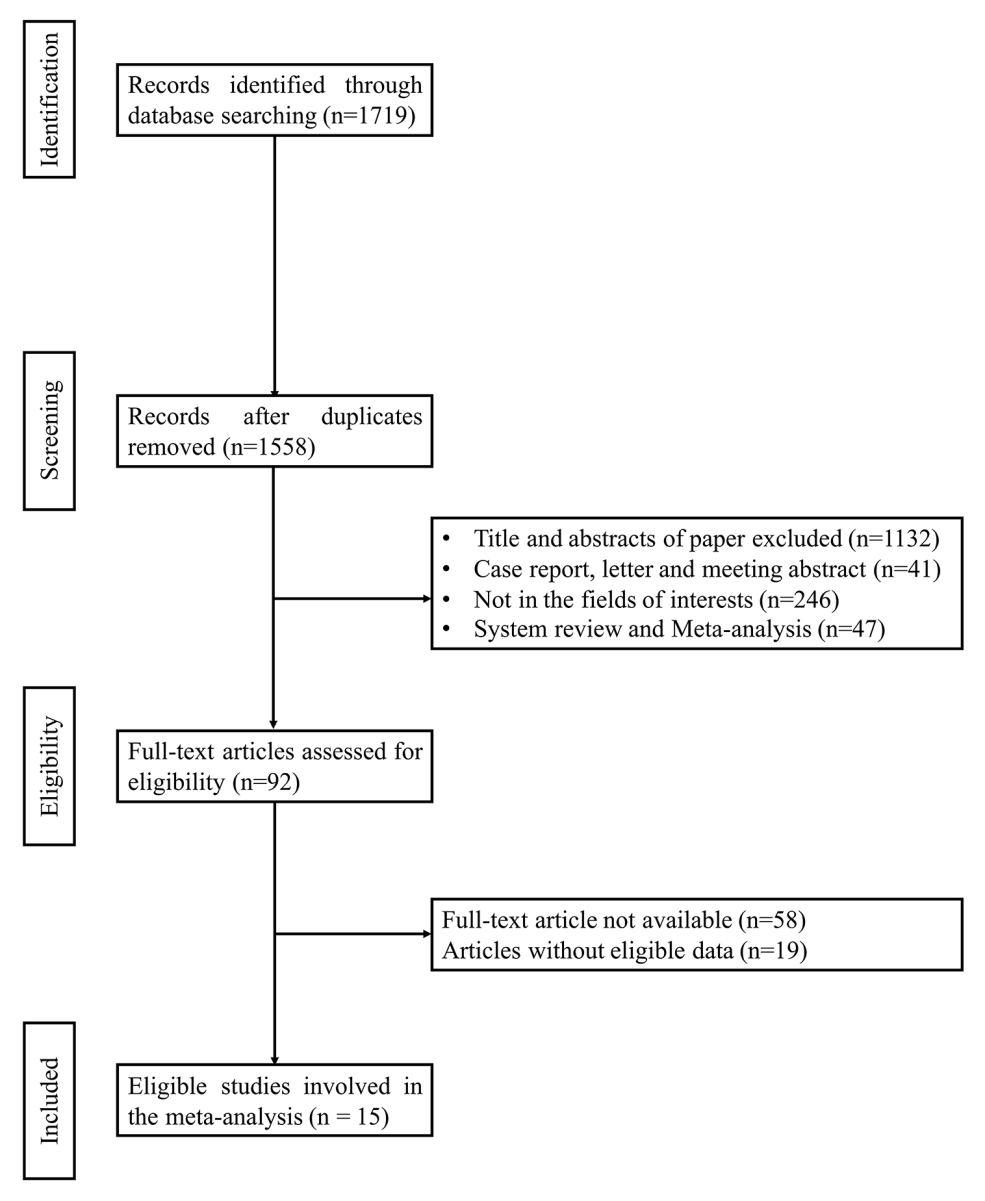
Flow chart of selection process to identify studies eligible for pooling.

**Figure 2:**
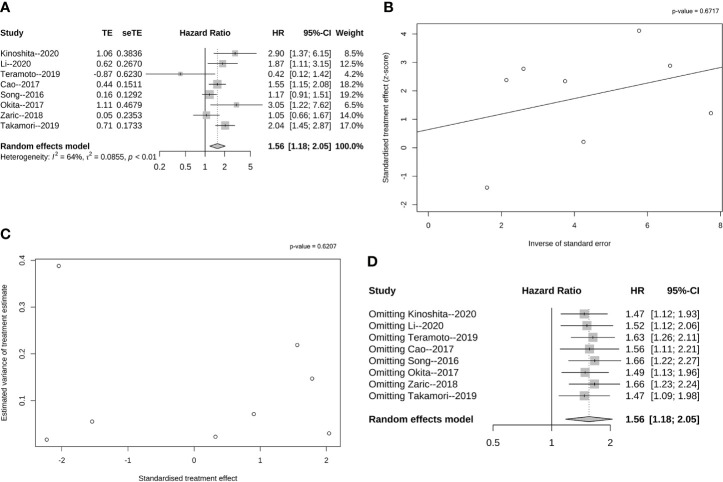
Pooled analysis of DFS according to PD-L1 expression. **(A)** Forest plot of HR for the association between PD-L1 expression and DFS with early stage resected non-small cell lung cancer; **(B)** Funnel graph of potential publication bias of HR for DFS in the eligible studies by Egger’s test; **(C)** Funnel graph of potential publication bias of HR for DFS in the eligible studies by Begg’s test. **(D)** Sensitivity analysis for DFS *via* elimination of each study in turn.

**Figure 3:**
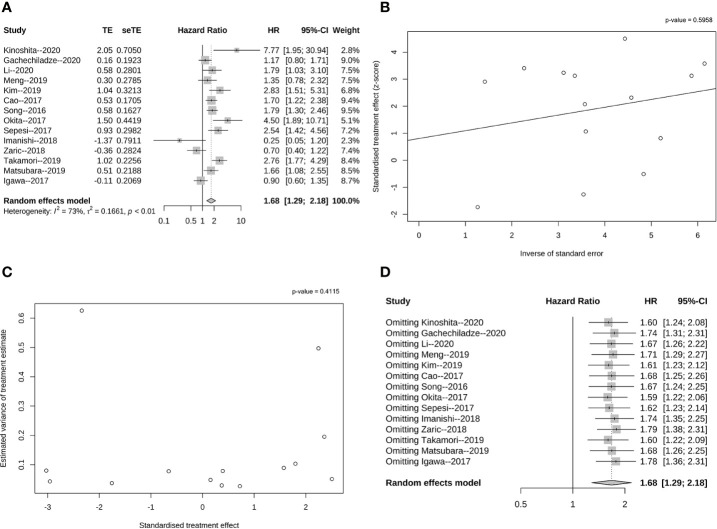
Pooled analysis of OS according to PD-L1 expression. **(A)** Forrest plot of HR for the association between PD-L1 expression and OS with early stage resected non-small cell lung cancer; **(B)** Funnel graph of potential publication bias of HR for OS in the eligible studies by Egger’s test; **(C)** Funnel graph of potential publication bias of HR for OS in the eligible studies by Begg’s test. **(D)** Sensitivity analysis for OS *via* elimination of each study in turn.

We identified 1,719 potentially relevant records through our search. A total of 1,558 studies were excluded after reviewing the title and abstract as their research contents failed to meet our inclusion criteria. Subsequently, the full texts of 92 articles were carefully screened, and a total of 15 studies (18–32) were found eligible for the final analysis. **Figure 1** summarizes the flow chart depicting the process followed for study selection. No article was excluded by methodological quality and risk of bias and sensitivity analysis for significant heterogeneity (**Figures 2D**, **3D**).

In all articles, resectability was a necessary intervention. All 15 studies were retrospective and published between 2016 and 2020. Fourteen of 15 studies recorded OS data, while only eight out of 15 studies presented only DFS information. Overall, 15 studies comprising of 3,790 patients were included in the pooled analysis and, all selected studies used immunohistochemistry or H-score assays to evaluate PD-L1 expression in tumor cells and/or tumor-infiltrating lymphocytes (TILs). **Table 1** summarized the characteristics of all involved studies, including peri-operative therapy and PD-L1 detection information.

**Table 1:** Summary characteristics of included studies.

Author	Years	Region	NOS(star) /NICE	Cancer type	Perioperative adjuvant therapy	Stage	Method	PD-L1 expression			Number	Antibody				Outcome
								Cut-off values	Negative	Positive		Company	Source	Type	Clone	
Kinoshita et al. (18)	2020	Japan	7	AD	NP	I	IHC	1%	155	48	203	Spring Biosclence, USA	Mouse	MAB	SP142	OS PFS
Gachechiladze et al. (19)	2020	Europe	8	NSCLC	12% neoadjuvant CT or CRT; 31% adjuvant CT; 18% adjuvant RT	I-III	IHC	NA	755	109	864	NA	Rabbit	PAB	SP263	OS
Li et al. (20)	2020	China	6	NSCLC	NP	I-III	IHC	5%	56	31	87	NA	Rabbit	DAB	66248-1-Ig	OS PFS
Meng et al. (21)	2019	China	7	NSCLC	29% adjuvant CT; 22% adjuvant CRT	I-III	IHC	10%	NA	NA	197	Abcam, USA	Rabbit	NA	28-8	OS
Kim et al. (22)	2019	Korea	7	AD	NP	I-III	IHC	5%	241	60	301	Spring Bioscience, USA	Rabbit	DAB	SP142	OS
Teramoto et al. (23)	2019	Japan	6	NSCLC	25% adjuvant CT	I-III	IHC	50%	104	21	125	Cell Signaling Technology, USA	Mouse	DAB	E1L3N	PFS
Cao et al. (24)	2017	China	7	NSCLC	61% adjuvant therapy*	I-III	IHC	50%	283	81	364	Cell Signaling Technology, USA	Mouse	DAB	EIL3N	OS PFS
Song et al. (25)	2016	China	7	AD	70% adjuvant therapy*	I-III	IHC	5%	199	186	385	Proteintech Group Inc.,USA	Rabbit	NA	66248-1-Ig	OS PFS
Okita et al. (26)	2017	Japan	6	NSCLC	NP	I-III	H-socre	100	78	13	91	Spring Biosclence, USA	Mouse	MAB	SP142	OS PFS
Sepesi et al. (27)	2017	America	7	NSCLC	21% adjuvant therapy*	I	IHC	4.69%	87	26	113	Cell Signaling Technology,USA	NA	NA	E1L3N	OS
Imanishi et al. (28)	2018	Japan	6	NSCLC	35% adjuvant therapy*	I-III	IHC	15%	16	10	26	Cell Signaling Technology,Japan	Rabbit	NA	E1L3N	OS
Zaric et al. (29)	2018	Austria	7	AD	46% adjuvant CT;7% adjuvant RT	I-III	IHC	1%	102	59	161	Cell Signaling	Rabbit	NA	E1L3N	OS PFS
Takamori et al. (30)	2019	Japan	8	AD	NP	I-III	IHC	1%	287	146	433	Spring Bioscience,USA	NA	NA	SP142	OS PFS
Matsubara et al. (31)	2019	Japan	7	SCC	NP	I-III	IHC	5%	134	77	211	Spring Bioscience, USA	Rabbit	DAB	SP142	OS
Igawa et al. (32)	2017	Japan	7	NSCLC	15% adjuvant therapy*	I-III	H-socre	100	109	120	229	Ventana Medical Systems, USA	Rabbit	PAB	SP263	OS

NSCLC, non-small cell lung cancer; AD, adenocarcinoma; SCC, squamous cell carcinoma; IHC, immunohistochemistry; OS, overall survival; DFS, disease-free survival; NA, not available; NP, not provided; CT, chemotherapy; CRT, chemo/radiotherapy; RT, radiotherapy.

*The exact means of therapy (CT, RT or CRT) is not clearly mentioned.

As shown in **Figure 2A**, the overall pooled HR indicated that the high PD-L1 expression was related to a significantly shorter DFS (HR = 1.56, 95% CI: 1.18–2.05, p < 0.01). Further analysis demonstrated heterogeneity among the eight studies (I² = 64%, p<< 0.01), and we hence performed Egger’s and Begg’s tests. According to Egger’s test (p = 0.671, **Figure 2B**) and Begg’s test (p = 0.620, **Figure 2C**), no publication bias influenced the HRs for DFS. Additionally, this confirmed that a negative correlation existed between PD-L1 expression and DFS in the case of resectable NSCLC patients.

In NSCLC patients, the positive expression of PD-L1 on tumor tissues was associated with significantly poorer OS when compared to those indicating negative PD-L1 expression (HR = 1.68, 95% CI: 1.29–2.18, p < 0.01, **Figure 3A**). Furthermore, heterogeneity was observed among the 15 studies (I² = 73%, P < 0.01). However, Egger’s (p = 0.595, **Figure 3B**) and Begg’s tests (p = 0.411, **Figure 3C**) demonstrated no publication bias influencing the HRs for OS, confirming the negative correlation of PD-L1 expression and OS for resectable NSCLC patients.

The correlation of PD-L1 expression and clinical characteristics was further analyzed, including gender, histology, tumor stage, smoking status, and lymph node metastasis. We observed that PD-L1 expression was significantly associated with gender (male *vs*. female: OR = 1.27, 95% CI:1.01–1.59, p = 0.038), histology (ADC *vs*. SCC: OR = 0.54, 95% CI:0.38–0.77, p = 0.001), TNM stage (I *vs*. II–III: OR = 0.45, 95% CI:0.34–0.60, p = 0.000), smoking status (Yes *vs* No: OR = 1.43,95% CI:1.14–1.80, p = 0.002) and lymph node metastasis (N+ *vs* N−:OR = 1.97, 95%CI:1.26–3.08, p = 0.003) (**Table 2**).

**Table 2:** The relationship between PD-L1 expression and clinicopathologic features.

Clinicopathological characteristics	No. of studies	Heterogeneity		OR	95%CI	P-value
		P-value	I² (%)			
**Gender (male vs. female)**	8	0.023	16.2	1.27	1.01-1.59	0.038
**Histology (ADC vs. SCC)**	5	0.044	8.12	0.54	0.38-0.77	0.001
**TNM stage (I vs. II-III)**	5	0.000	40.2	0.45	0.34-0.60	0.000
**Smoking status (yes vs. no)**	8	0.008	18.9	1.43	1.14-1.80	0.002
**Lymph node metastasis (N+ vs. N0)**	3	0.425	1.71	1.97	1.26-3.08	0.003

AD, adenocarcinoma; SCC, squamous cell carcinoma; OR, odds ratio; 95%CI, 95%confidence intervals.

In addition, we also investigated the different cutoff values of PD-L1 expression on the survival. We found that PD-L1 expression at 5% cutoff value was not correlated to DFS (HR = 1.39, 95% CI: 0.89–2.17, p = 0.142) (**Supplementary Data 4**), but indicated a significantly worse OS (HR = 1.93, 95% CI: 1.57–2.36, p = 0.000) (**Supplementary Data 5**). However, PD-L1 expression at 1% cutoff value was correlated with a poor DFS (HR = 1.76, 95% CI: 1.03–3.02, p = 0.040) (**Supplementary Data 6**) but not for OS (HR = 2.19, 95% CI: 0.66–7.22, p = 0.199) (**Supplementary Data 7**). We propose that the discrepancy for these findings may be attributed to limited data and non-uniform PD-L1 detection platforms.

## Discussion

Immune checkpoint inhibitors targeting PD-1/PD-L1 have improved survival in patients with advanced NSCLC in second- and first-line settings (33–37). The PACIFIC trial reported a DFS benefit in patients with locally advanced, unresectable stage III NSCLC who received durvalumab consolidation therapy (38). Owing to the success of immune checkpoint inhibitors in advanced NSCLC, these agents are currently under investigation in the neo- or adjuvant setting for resected NSCLC patients (39–42) Therefore, it is important to understand the impact of PD-1/PD-L1 expression on the prognosis of early stage, resected NSCLC patients. Several studies have demonstrated that PD-L1 is a biomarker indicating poor prognosis and survival in advanced-stage NSCLC patients. However, as reported by previous studies, the expression of PD-L1 on the prognosis of early stage resected NSCLC remains controversial. A comprehensive analysis is required to integrate all available data and provide further insight on this issue.

By summarizing the data available from included studies, our results confirmed that the PD-L1 expression indicates an unfavorable prognosis in early stage resected NSCLC as well. Our conclusion that PD-L1 positive or high expression indicated a significantly inferior OS in early stage resected NSCLC, is consistent with the previous analysis for all stages or advanced stage NSCLC patients (9–11). Additionally, we demonstrate that DFS, which was not explored by the previous studies, is also negatively correlated with PD-L1 expression in early stage resected NSCLC. Lastly, based on subgroup analysis, we observed that the PD-L1 expression was associated with gender, histology, tumor stage, smoking status, and lymph node metastasis.

Our analysis provided evidence to support that the PD-L1 expression may have prognostic value in predicting survival of patients with resected NSCLC. However, there were several limitations to our study. Firstly, this was based on a retrospective analysis. A prospective analysis is required to further clarify these issues. Secondly, 12 out of 15 included studies were performed in Asian population. Although it was not our intention to ignore the data from non-Asian population, the lack of data from non-Asian population is still a limitation for our meta-analysis. Therefore, future investigation should focus more on the PD-L1 expression in early stage non-Asian NSCLC patients. Thirdly, the different adjuvant treatment strategies post-surgery and follow-up also influence the survival of patients with NSCLC undergoing resection, which could have influenced the analysis. Moreover, the cutoff value of the defined PD-L1 expression was rather different among the included studies. We have to categorize high (positive) and low (negative) PD-L1 expression and study the correlation between PD-L1 expression and post-operative survival. Finally, the platform and antibody of PD-L1 detection was not uniform either in the included studies. We have mentioned this issue in **Table 1**.

## Conclusions

In conclusion, our meta-analysis suggests that PD-L1 expression indicates an unfavorable prognosis in early stage resected NSCLC patients. In the adjuvant settings resected NSCLC, the role of individualized anti-PD-L1/PD-1 immunotherapy merits further investigated.

## Data Availability Statement

The original contributions presented in the study are included in the article/**Supplementary Material**. Further inquiries can be directed to the corresponding authors.

## Author Contributions

TS, SZ, and HG retrieved and analyzed all of the data in the study. XFL, YW, XL, and SKZ revised the manuscript for important intellectual contents. LP and DH reviewed and edited the manuscript. SX, ZL, and LP designed, checked, and supervised all study processes. All authors contributed to the article and approved the submitted version.

## Funding

The present study was funded by the National Natural Science Foundation of China (No. 81772464), Natural Science Foundation of Zhejiang Province, China (No. LY19H160041), Shanghai Science and Technology Commission Guidance Projects (No. 18411968200), Medical-Engineering Joint Funds from Shanghai Jiao Tong University (No. YG2017MS81) and Shanghai Chest Hospital Project of Collaborative Innovation (No. YJXT20190209).

## Conflict of Interest

The authors declare that the research was conducted in the absence of any commercial or financial relationships that could be construed as a potential conflict of interest.

## Supplementary Material

Supplementary Data Sheet 1 The PRISMA checklist of the manuscript.

Supplementary Data Sheet 2 The search strategy of the manuscript.

Supplementary Data Sheet 3 The data of sensitivity analyses.

Supplementary Data Sheet 4 Forest plot of HR for the association between PD-L1 expression and DFS with early stage resected non-small cell lung cancer. (PD-L1 expression at 5% cutoff value).

Supplementary Data Sheet 5 Forest plot of HR for the association between PD-L1 expression and OS with early stage resected non-small cell lung cancer. (PD-L1 expression at 5% cutoff value).

Supplementary Data Sheet 6 Forest plot of HR for the association between PD-L1 expression and DFS with early stage resected non-small cell lung cancer. (PD-L1 expression at 1% cutoff value).

Supplementary Data Sheet 7 Forest plot of HR for the association between PD-L1 expression and OS with early stage resected non-small cell lung cancer. (PD-L1 expression at 1% cutoff value).
