# Supplementary material for: The Impact of Programmed Death-Ligand 1 Expression on the Prognosis of Early Stage Resected Non-Small Cell Lung Cancer: A Meta-Analysis of Literatures
Source: Front Oncol. 2021 Feb 23;11:567978. doi: 10.3389/fonc.2021.567978 (PMC7940546; doi:10.3389/fonc.2021.567978)
Supplement: Supplementary Data Sheet 2 — The search strategy of the manuscript. [file DataSheet_2.docx]

Search strategy in Pubmed (2020-7-23)

| # | Query |
| --- | --- |
| #1 | ("B7-H1 Antigen"[Mesh]) OR (((((((((((((B7 H1 Antigen[Title/Abstract]) OR (Programmed Cell Death 1 Ligand 1[Title/Abstract])) OR (B7-H1 Immune Costimulatory Protein[Title/Abstract])) OR (B7 H1 Immune Costimulatory Protein[Title/Abstract])) OR (PD-L1 Costimulatory Protein[Title/Abstract])) OR (Costimulatory Protein, PD-L1[Title/Abstract])) OR (PD L1 Costimulatory Protein[Title/Abstract])) OR (Programmed Cell Death 1 Ligand 1 Protein[Title/Abstract])) OR (CD274 Antigen[Title/Abstract])) OR (Antigen, CD274[Title/Abstract])) OR (Antigens, CD274[Title/Abstract])) OR (CD274 Antigens[Title/Abstract])) OR (B7H1 Immune Costimulatory Protein[Title/Abstract])) |
| #2 | ("Carcinoma, Non-Small-Cell Lung"[Mesh]) OR ((((((((((Carcinoma, Non Small Cell Lung[Title/Abstract]) OR Carcinomas, Non-Small-Cell Lung[Title/Abstract]) OR Lung Carcinoma, Non-Small-Cell[Title/Abstract]) OR Lung Carcinomas, Non-Small-Cell[Title/Abstract])OR Non-Small-Cell Lung Carcinomas[Title/Abstract]) OR Nonsmall Cell Lung Cancer[Title/Abstract]) OR Non-Small-Cell Lung Carcinoma[Title/Abstract]) OR Non Small Cell Lung Carcinoma[Title/Abstract]) OR Carcinoma, Non-Small Cell Lung[Title/Abstract]) OR Non-Small Cell Lung Cancer[Title/Abstract]) |
| #3 | (prognosis[MeSH:noexp] OR diagnosed[Title/Abstract] OR cohort*[Title/Abstract] OR cohort effect[MeSH Term] OR cohort studies[MeSH:noexp] OR predictor*[Title/Abstract] OR death[Title/Abstract] OR "models, statistical"[MeSH Term]) |
| #4 | #1AND#2AND#3 |

Search strategy in Cochrane library (2020-7-23)

| # | Query |
| --- | --- |
| #1 | MeSH descriptor: [Carcinoma, Non-Small-Cell Lung] explode all trees |
| #2 | (Carcinoma, Non-Small Cell Lung):ti,ab,kw OR (Non-Small Cell Lung Cancer):ti,ab,kw OR (Carcinomas, Non-Small-Cell Lung):ti,ab,kw OR (Non Small Cell Lung Carcinoma):ti,ab,kw OR (Lung Carcinomas, Non-Small-Cell):ti,ab,kw |
| #3 | (Carcinoma, Non Small Cell Lung):ti,ab,kw OR (Non-Small-Cell Lung Carcinomas):ti,ab,kw OR (Non-Small-Cell Lung Carcinoma):ti,ab,kw OR (Nonsmall Cell Lung Cancer):ti,ab,kw OR (Lung Carcinoma, Non-Small-Cell):ti,ab,kw |
| #4 | (#1OR#2OR#3) |
| #5 | MeSH descriptor: [B7-H1 Antigen] explode all trees |
| #6 | (B7 H1 Immune Costimulatory Protein):ti,ab,kw OR (Programmed Cell Death 1 Ligand 1):ti,ab,kw OR (CD274 Antigens):ti,ab,kw OR (Costimulatory Protein, PD-L1):ti,ab,kw OR (Antigens, CD274):ti,ab,kw |
| #7 | (B7 H1 Antigen):ti,ab,kw OR (Antigen, CD274):ti,ab,kw OR (B7H1 Immune Costimulatory Protein):ti,ab,kw OR (PD L1 Costimulatory Protein):ti,ab,kw OR (Programmed Cell Death 1 Ligand 1 Protein):ti,ab,kw |
| #8 | (PD-L1 Costimulatory Protein):ti,ab,kw OR (CD274 Antigen):ti,ab,kw |
| #9 | (#5OR#6OR#7OR#8) |
| #10 | (#4AND#9) |
| #11 | MeSH descriptor: [Prognosis] explode all trees |
| #12 | (Factors, Prognostic):ti,ab,kw OR (Prognostic Factors):ti,ab,kw OR (Prognostic Factor):ti,ab,kw OR (Factor, Prognostic):ti,ab,kw OR (Prognoses):ti,ab,kw |
| #13 | (#11OR#12) |
| #14 | (#13AND#10) |

Search strategy in Embase (2020-7-23)

| # | Query |
| --- | --- |
| #1 | 'non small cell lung cancer'/exp OR 'bronchial non small cell cancer':ab,ti OR 'bronchial non small cell carcinoma':ab,ti OR 'carcinoma, non-small-cell lung':ab,ti OR 'lung cancer, non small cell':ab,ti OR 'lung non small cell cancer':ab,ti OR 'lung non small cell carcinoma':ab,ti OR 'non small cell bronchial cancer':ab,ti OR 'non small cell cancer, lung':ab,ti OR 'non small cell lung carcinoma':ab,ti OR 'non small cell pulmonary cancer':ab,ti OR 'non small cell pulmonary carcinoma':ab,ti OR 'pulmonary non small cell cancer':ab,ti OR 'pulmonary non small cell carcinoma':ab,ti |
| #2 | 'programmed death 1 ligand 1'/exp OR 'antigen b7 h1':ab,ti OR 'antigen b7h1':ab,ti OR 'antigen cd274':ab,ti OR 'antigens, cd274':ab,ti OR 'b7 h1 antigen':ab,ti OR 'b7 h1 protein':ab,ti OR 'b7 homolog 1 protein':ab,ti OR 'b7-h1 antigen':ab,ti OR 'b7h1 antigen':ab,ti OR 'b7h1 protein':ab,ti OR 'cd274 antigen':ab,ti OR 'cd274 antigens':ab,ti OR 'pdcd1 ligand 1':ab,ti OR 'pdcd1lg1 protein':ab,ti OR 'programmed cell death 1 ligand 1':ab,ti OR 'programmed death 1 ligand 1 protein':ab,ti OR 'programmed death ligand 1':ab,ti OR 'protein b7 h1':ab,ti OR 'protein b7h1':ab,ti OR 'protein pdcd1lg1':ab,ti |
| #3 | 'prognosis'/exp OR 'follow-up.mp.' OR 'prognos:.tw.' OR 'ep.fs.' OR 'survival.tw.' OR 'outcome.tw.' OR 'follow-up.mp.' |
| #4 | #1AND#2AND#3 |
