# Supplementary material for: The Impact of Programmed Death-Ligand 1 Expression on the Prognosis of Early Stage Resected Non-Small Cell Lung Cancer: A Meta-Analysis of Literatures
Source: Front Oncol. 2021 Feb 23;11:567978. doi: 10.3389/fonc.2021.567978 (PMC7940546; doi:10.3389/fonc.2021.567978)
Supplement: Supplementary Data Sheet 3 — The data of sensitivity analyses. [file DataSheet_3.docx]

| Linear | regression | test | of | funnel | plot | asymmetry |
| --- | --- | --- | --- | --- | --- | --- |

| data: | mr |
| --- | --- |

| t | = | 0.54497, | df | = | 12, | p-value | = 0.5958 |
| --- | --- | --- | --- | --- | --- | --- | --- |

| alternative | hypothesis: | asymmetry | in | funnel | plot |
| --- | --- | --- | --- | --- | --- |

| sample | estimates: |
| --- | --- |

| bias | se.bias | intercept |
| --- | --- | --- |

| 0.8060947 | 1.4791611 | 0.2890590 |
| --- | --- | --- |

| Rank | correlation | test | of | funnel | plot | asymmetry |
| --- | --- | --- | --- | --- | --- | --- |

| data: | mr |
| --- | --- |

| z = | 0.82117, | p-value | = | 0.4115 |
| --- | --- | --- | --- | --- |

| alternative | hypothesis: | asymmetry | in | funnel | plot |
| --- | --- | --- | --- | --- | --- |

| sample | estimates: |
| --- | --- |

| ks | se.ks |
| --- | --- |

| 15.00000 | 18.26655 |
| --- | --- |

| Linear | regression | test | of | funnel | plot | asymmetry |
| --- | --- | --- | --- | --- | --- | --- |

| data: | mr |
| --- | --- |

| t | = | 0.44535, | df | = | 6, | p-value | = 0.6717 |
| --- | --- | --- | --- | --- | --- | --- | --- |

| alternative | hypothesis: | asymmetry | in | funnel | plot |
| --- | --- | --- | --- | --- | --- |

| sample | estimates: |
| --- | --- |

| bias | se.bias | intercept |
| --- | --- | --- |

| 0.6397531 | 1.4365055 | 0.2726108 |
| --- | --- | --- |

PFS_Egger’s test

OS_Egger’s test


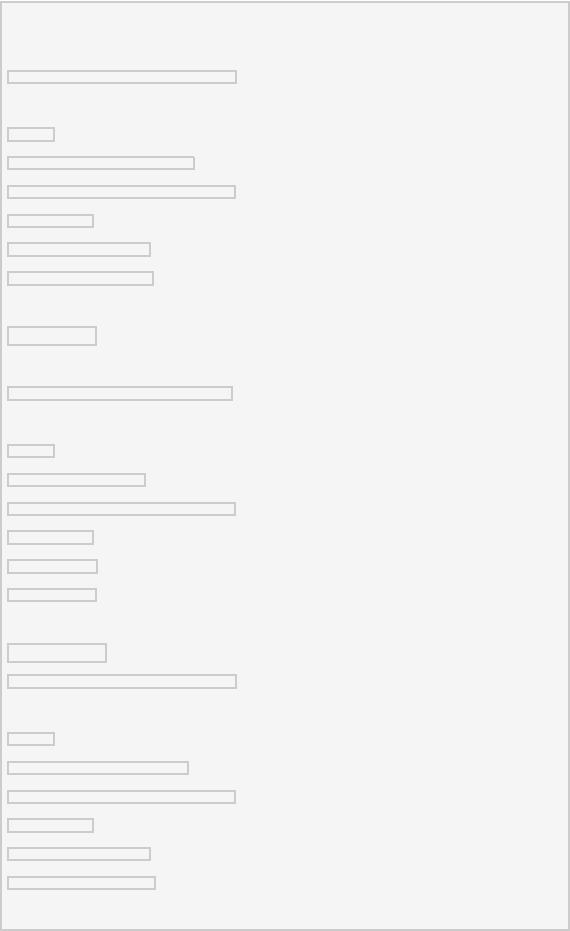


OS_Begg’s test

| Rank | correlation | test | of | funnel | plot | asymmetry |
| --- | --- | --- | --- | --- | --- | --- |

| data: | mr |
| --- | --- |

| z | = | 0.49487, | p-value | = | 0.6207 |
| --- | --- | --- | --- | --- | --- |

| alternative | hypothesis: | asymmetry | in | funnel | plot |
| --- | --- | --- | --- | --- | --- |

| sample | estimates: |
| --- | --- |

|  | ks | se.ks |
| --- | --- | --- |

| 4.000000 | 8.082904 |
| --- | --- |

PFS_Begg’s test
